# Depressive symptoms and 5-year incident metabolic syndrome among older adults

**DOI:** 10.1038/s41598-021-94503-y

**Published:** 2021-07-21

**Authors:** Qian Wu, Yi-Ying Hua, Qing-Hua Ma, Yong Xu, Xing Chen, Chen-Wei Pan

**Affiliations:** 1 School of Public Health, Medical College of Soochow University, 199 Ren Ai Road, Suzhou, 215123 China; 2 The 3Rd People’s Hospital of Xiangcheng District, Suzhou, China; 3 Department of Children Health Care, Affiliated Suzhou Hospital of Nanjing Medical University, No.26, Dao Qian Road, Suzhou, 215000 China

**Keywords:** Psychology, Endocrinology, Risk factors

## Abstract

Little is known regarding the association between depressive symptoms and metabolic syndrome (MetS) among older Chinese adults. This study aimed to examine the association of depressive symptoms with MetS and its components among Chinese elderly. Based on whether they showed depressive symptoms at baseline, 262 age-gender-matched participants from a community-based cohort study were included. The presence of depressive symptoms was measured using the nine-item Patient Health Questionnaire (PHQ-9). MetS was defined according to the Adult Treatment Panel III of the National Cholesterol Education Program. Linear regression and logistic regression analyses were performed to assess associations of depressive symptoms with MetS and its components. The incidence of MetS among the participants with depressive symptoms at baseline was 15.27% (20/131). The association of the presence of depressive symptoms with MetS was significant (odds ratio [OR] = 2.53, 95% confidence intervals [CI] = 1.07, 5.95). There was a negative association between depressive symptoms and hypertension (OR = 0.04, 95% CI = 0.002, 0.98). The change in mean arterial pressure varies approximately 1.03 mmHg with a 1-point change in PHQ-9 score. In this study, baseline depressive symptoms were associated with subsequent MetS. The presence of depressive symptoms was negatively associated with elevated mean arterial pressure.

## Introduction

Metabolic syndrome (MetS) is one of the prevalent concerns to global public health^1,2^. It is characterized by a clustering of cardiovascular risk factors including low level of high-density lipoprotein cholesterol (HDL-C), abdominal obesity, high blood pressure (BP), high level of triglycerides (TG), and elevated fasting plasma glucose (FPG) or diabetes. Current literatures have acknowledged that there is a bidirectional association of mental health with physical health^3,4^. Modern Chinese rural elderly people are facing much severer mental and physical challenge than ever^5^. Various factors such as aging^6^ and chronic disease^7^ which put a detrimental effect on mental health led to bad outcomes including the risk of disorders^8^ and mortality^9^ of the elderly. It has demonstrated that depression is one of the most common psychiatric illnesses in later life, with a prevalence of 4.5–37.4% in different studies^10^. There is substantial evidence to support that depression is also linked to cardiovascular disease^11^ and diabetes^12^. Hence, it’s reasonable to hypothesize that there is a relationship between depression and MetS.

In China, the development of MetS has risen rapidly, with the prevalence of 33.9% (31.0% in male and 36.8% in female)^13^. Previous studies have demonstrated the association of MetS with cognitive impairment^14^, dementia, and Alzheimer’s disease^15^. A recent system review reported that the depression was associated with 1.34 times the odds of developing the MetS^16^. To date, some cross-sectional studies indicated that depression or the presence of depressive symptoms was associated with MetS^17^ and the components of MetS^18^, whereas some studies did not detect such an association^19,20^. Some lifestyle habits were observed to contribute to the subsequent MetS^21^. However, few studies have assessed the relationship among Chinese^22^ and most of the longitudinal research is carried in developed countries or areas^16,23^. In East Asia, a Korean research^24^ revealed a positive correlation between depression trajectory and MetS in a retrospective cohort, which still remained a lack of control over demographic characteristics that may confuse the results.

It’s well known that lifestyles and living habits vary widely across geographical settings and different cultural backgrounds. Unique culture and lifestyle could alter the real association between depressive symptoms and MetS. Thus, the impact of different cultural differences on diseases is worthy of further exploration.

Accordingly, the aim of this study was to assess the association of initial depressive symptoms with subsequent MetS in Chinese adults aged 60 years or older, and further examine the extent to which potential explanatory factors impact the incidence of MetS in older people with or without depressive symptoms.

## Methods

### Study population

Data of this study were from the Weitang Geriatric Diseases study, which is a community-based cohort study conducted in Weitang town among adults aged 60 years and above in Suzhou, China. More detailed information of the study protocols has been described elsewhere^25^. According to the official record, 6,030 adults aged 60 years or older received an invitation letter. Participants were deemed eligible to participate in further study if (1) he or she had not migrated from the residential address; (2) residents had been living in the town for longer than six months; (3) he or she stayed alive. Finally, 4579 participants completed both interviewer-administered questionnaires and clinical examinations. The baseline investigation was conducted in 2014 and the follow-up investigation was carried out five years later.

**Figure 1:**
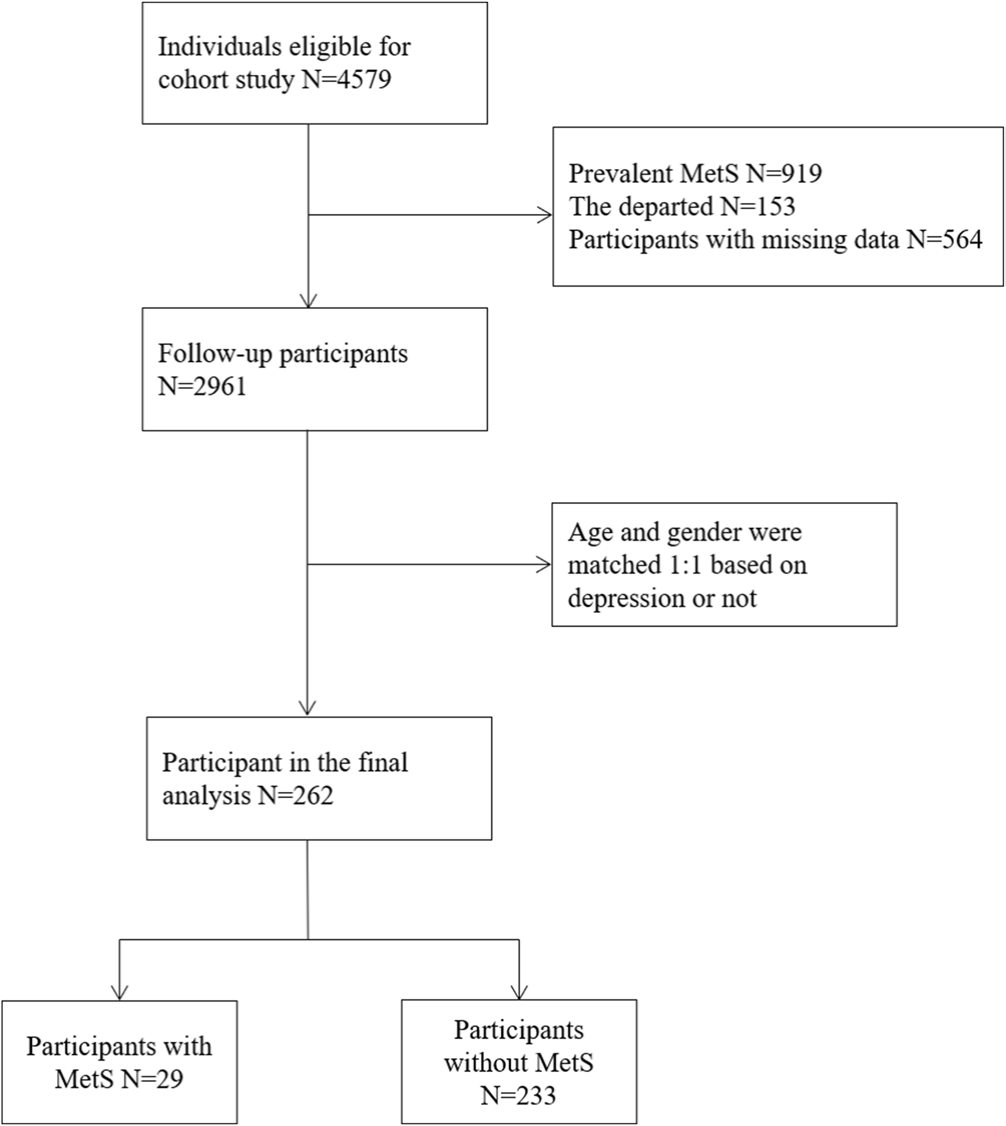
Flow diagram showing the screening of study participants. *MetS* metabolic syndrome.

Figure 1 describes the selection of participants included and excluded in the current analysis. We screened out 919 participants who had MetS at baseline, 153 participants who had departed and 564 participants with incomplete data. Of the remaining 2,961 participants, we found that a large discrepancy in age and gender between participants with or without the presence of depressive symptoms. Therefore, each participant with depressive symptoms was matched with one non-depressive counterpart based on the strategy of the same gender and same age, namely 131 participants with the presence of depressive symptoms and 131 were free of depressive symptoms.

All participants provided written informed consent before data collection. The study was undertaken in agreement with the Declaration of Helsinki and approved by the Institutional Review Board of Soochow University.

### Assessment of depressive symptoms

The nine-item Patient Health Questionnaire (PHQ-9), a widely utilized screening tool for depression and depressive symptoms in clinical settings^26^, was used to assess depressive symptoms at baseline. Items on the scale correspond to the criteria of Diagnostic and Statistical Manual of Mental Disorders (DSM, Fourth Edition)^27^. Eight items of the PHQ-9 evaluate how often they suffered from the depressive symptoms over the past two weeks, and the last item is about the frequency of thoughts that hurt themselves during the past two weeks. The response options are on a four-point Likert scale (0 = not at all, 1 = several days, 2 = more than half of the days, and 3 = nearly every day), and higher scores denote higher levels of depressive symptoms. A score of 5 or higher is indicative of having depressive symptoms^28^.

### Measurement of MetS and its components

MetS was defined according to the Adult Treatment Panel III of the National Cholesterol Education Program (ATP III-NCEP)^29^. Participants were considered as having MetS if they had three or more of the following conditions: (1) overweight or obesity (body mass index ≥ 25 kg/m^2^)^30^; (2) BP ≥ 130/85 mmHg or history of antihypertensive treatment; (3) the serum level of TG > 150 mg/dL (1.69 mmol/L); (4) HDL-C < 40 mg/dL in men or < 50 mg/dL in women; (5) FPG ≥ 7.0 mmol/L or previously confirmed diagnosis of diabetes mellitus.

### Assessment of covariates

Covariates were included as possible confounding variables affecting the metabolic abnormalities or depression^31–36^. All covariates included in the model were collected at baseline. Information of socio-demographic characteristics (i.e., age, gender, marital status, and education level) was collected through the pre-designed questionnaire. Marital status was divided into living ‘with’ or ‘without’ spouse. The education level was divided into two groups, one was participants who have completed elementary education or junior high school education or senior high school education or the university and above. The other group, namely ‘no formal education’ referred to participants who have completed none of them. Lifestyle-related factors were also recorded such as smoking (present/past/never), alcohol consumption (drinkers/non-drinkers), tea consumption (drinkers/non-drinkers), dietary patterns (normal/vegetarian) and physical activity (yes/no). The alcohol consumption referred to the answers of ‘whether the participants had drunk alcohol drink in the past three months’. Normal diet referred to participants who ate meat or fish. The histories of cardiovascular diseases including heart disease (yes/no) and stroke (yes/no) were self-reported by participants.

### Statistical analysis

Given the high correlation of systolic and diastolic blood pressure, we rescaled them into a single measure of mean arterial pressure (MAP), using the following standard Eq. (1)^37^: $\mathrm{MAP}=\frac{(2*{\varvec{P}}\boldsymbol{ } {\text{diastolic}}+{\varvec{P}}\boldsymbol{ } {\text{systolic}})}{3}$.

Categorical variables of participants with or without depressive symptoms were compared with a cross-tabulation, using Pearson’s χ^2^ test. The cumulative incidence of MetS was estimated as the number of cases divided by persons at risk only those who fully completed the follow-up (selected through the matching).

To estimate the associations of depressive symptoms (defined as the PHQ-9 scores as a continuous variable) with the change in each component of MetS, multivariate regression models were fitted, adjusting for education level, marital status, smoking, alcohol consumption, tea consumption, physical activity and heart disease. The model assumptions were checked. Variance infiltration factor (VIF) was used to identify the multicollinearity between the variables in each model. A VIF > 10.0 was considered significant multicollinearity in the regression model^38^. The change in each component is calculated as the results of follow-up clinical examination minus the results at baseline. Before proceeding to analyze the associations of depressive symptoms with the change in each component of MetS, we excluded subjects who met the clinical criteria for individual components of MetS at baseline.

To evaluate the extent to which the risk factors may explain the excess incidence of MetS in participants with depressive symptoms compared with those without depressive symptoms, we estimated the percentage of reduction in odds associated with adjustment for these factors based on the following formula: (R_a_-R_b_)/(R_a_-1) × 100, where R_a_ is the OR of MetS in the participants with depressive symptoms compared with their non-depressive counterparts in the crude model, and R_b_ is the OR in models after additional adjustment. Such method was applied in previous research^39^. In this study, the excess incidence referred to the incidence of MetS in participants with depressive symptoms compared to participants without depressive symptoms.

To test whether depressive symptoms were associated with MetS and each of its components, logistic regression models were performed and odds ratios (OR) and 95% confidence intervals (CI) were shown. Education level, marital status, smoking, alcohol consumption, tea consumption, physical activity and history of heart disease were included in the analytic models as covariates.

All statistical analyses were carried out using IBM SPSS Statistics for Windows (Version 22.0. Armonk, NY: IBM Corp., USA), and a *P* value of less than 0.05 was considered statistically significant.

## Results

Table 1 lists the demographic characteristics of study participants at baseline. The mean age of participants was 74.41 years (age range 63–90). Among them, the mean age in the oldest-old participants (> 80 years) was 84.01 ± 5.87 years compared with 70.70 ± 4.86 years in more young-old (≤ 80 years) participants. Among the samples, the incidence of MetS was 15.27% in participants with depressive symptoms at baseline and 6.9% in people who were free of depressive symptoms at baseline. People without depressive symptoms tended to live with spouse. Compared with the non-depressive counterparts group, people with depressive symptoms had less physical activity, higher consumption of tea, and higher prevalence of stroke.

**Table 1:** Characteristics of participants with or without the presence of depressive symptoms (N = 262).

Variables, n (%)	The presence of depressive symptoms		*P* value
	With (N = 131)	Without (N = 131)	
**Sex**			1.000
Male	37(28.24)	37(28.24)	
Female	94(71.86)	94(71.86)	
Onset of MetS	20 (15.27)	9 (6.87)	0.03
Primary education and higher	45 (34.35)	52 (39.69)	0.37
Living with spouse	83 (63.36)	101 (77.10)	0.02
Current smoker	19 (14.50)	22 (16.79)	0.63
Current alcohol drinker	16 (12.21)	18 (13.74)	0.71
Current tea drinker	21 (16.03)	34 (25.95)	0.049
Vegetarian	4 (3.05)	0 (0)	0.04
With physical activity	43 (32.82)	64 (48.85)	0.01
History of heart disease	17 (12.98)	14 (10.69)	0.57
History of stroke	7 (5.34)	0 (0)	0.01
Antihypertensive medications	63 (48.09)	68 (51.91)	0.54
Diagnosis of diabetes	7 (5.34)	5 (3.82)	0.36
Antidiabetic medications	4 (3.05)	4 (3.05)	0.41
Taking insulin	3 (2.29)	2 (1.53)	0.92
**Number of MetS components**			0.26
0	9	8	
1	66	76	
2	36	38	
3	16	8	
4	4	1	
5	0	0	

*MetS* metabolic syndrome.

The change in MAP varied approximately 1.03 mmHg with a 1-point increase in PHQ-9 score. The change in MAP was significantly associated with the presence of depressive symptoms (as a continuous variable) (*P* = 0.047), after adjusting for education level, marital status, smoking, alcohol consumption, tea consumption, physical activity and heart disease (Table 2). None of the variables showed significant collinearity with each other (all VIF < 10.0). Additionally, no significant association were observed between the depressive symptoms and different variations of risk factors numbers (data not shown).

**Table 2:** Associations of PHQ-9 score with change in individual components of MetS.

Change in individual components of MetS^a^	Model 1			Model 2		
	Beta^b^	95% CI	*P* value	Beta^b^	95% CI	*P* value
Change in BMI value	− 0.04	− 0.13, 0.05	0.41	− 0.06	− 0.16, 0.03	0.19
Change in MAP^c^	− 0.91	− 1.96, 0.13	0.08	− 1.03	− 2.04, − 0.02	0.047
Change in TG	− 0.002	− 0.02, 0.01	0.81	− 0.001	− 0.02, 0.01	0.89
Change in HDL-C	− 0.003	− 0.01, 0.01	0.46	− 0.003	− 0.01, 0.01	0.50
Change in FPG	0.004	− 0.01, 0.02	0.64	0.01	− 0.01, 0.02	0.37

*PHQ-9* the nine-item Patient Health Questionnaire, *MetS* metabolic syndrome, *CI* confidence interval, *BMI* body mass index, *MAP* mean arterial pressure, *TG* triglycerides, *HDL-C* high-density lipoprotein cholesterol, *FPG* fasting plasma glucose.

Model 1: crude model; Model 2: adjusted for education level, marital status, smoking, alcohol consumption, tea consumption, physical activity and heart disease.

^A^the change in individual components of MetS is calculated as the results of follow-up physical examination minus the results in the baseline.

^b^Unstandardized regression coefficient.

^c^MAP was calculated as $\mathrm{MAP}=\frac{(2*{\varvec{P}}\boldsymbol{ } {\text{diastolic}}+\mathbf{P}\boldsymbol{ } {\text{systolic}})}{3}$.

We estimated the reduction in odds of MetS associated with the presence of depressive symptoms with adjustment of MetS-related variables. Adjustment for education level, smoking, alcohol consumption or history of heart disease led to reduction in the excess incidence of MetS in participants with depressive symptoms by 4.17%, 4.86%, 0.69% or 0.69%, respectively. On the contrary, adjustment for marital status and physical activity increased the excess incidence of MetS by 6.25%, 5.56%, relatively (Table 3).

**Table 3:** Effect of potential explanatory factors on the excess incidence of MetS in participants with depressive symptoms at baseline compared with the non-depressive counterparts group.

Model^a^	OR	95% CI	*P* value	% reduction excess incidence^b^
1	2.44	1.07, 5.59	0.03	Reference
2	2.38	1.04, 5.50	0.04	4.17
3	2.53	1.10, 5.85	0.03	− 6.25
4	2.37	1.03, 5.44	0.04	4.86
5	2.43	1.06, 5.56	0.04	0.69
6	2.30	1.00, 5.29	0.05	9.72
7	2.52	1.09, 5.82	0.03	− 5.56
8	2.43	1.06, 5.57	0.04	0.69
9	2.53	1.07, 5.95	0.03	− 6.25

*MetS* metabolic syndrome, *OR* Odds ratio, *CI* confidence interval.

^a^Adjusted for the following variables: model 1: crude; model 2: education level; model 3: marital status; model 4: smoking; model 5: alcohol consumption; model 6: tea consumption; model 7: physical activity; model 8: heart disease; model 9: education level, marital status, smoking, alcohol consumption, tea consumption, physical activity and heart disease.

^b^% reduction in excess morbidity defined by the formula: (R_a_−R_b_)/(R_a_−1) × 100, where R_a_ is the OR of onset of MetS in participants with depressive symptoms at baseline compared with the non-depressive counterparts group adjusted in model 1 (crude model, reference) and R_b_ is the OR after additional adjustment for the variables in models 2 to 9.

**Figure 2:**
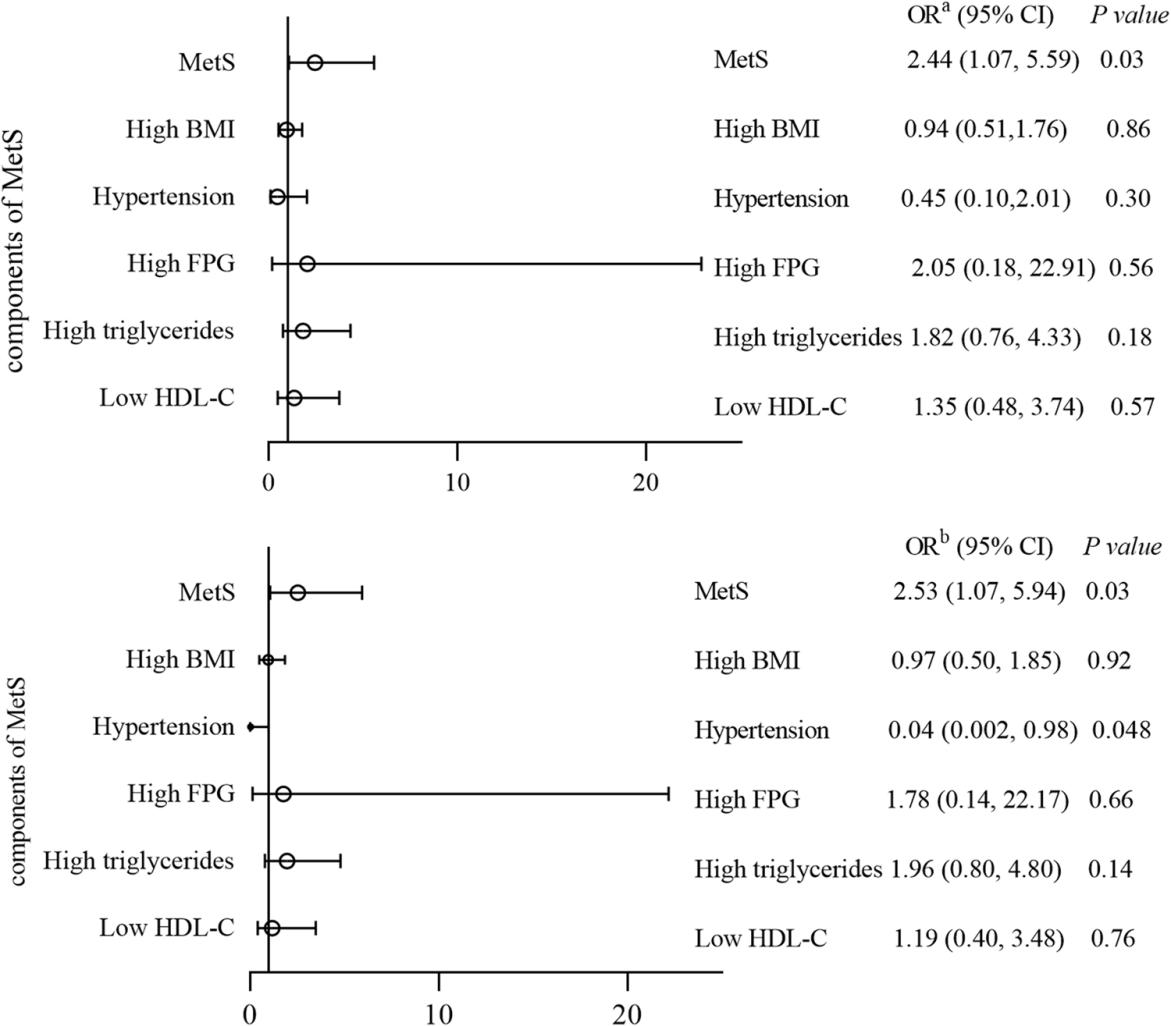
Relationship between depressive symptoms and metabolic components with or without multivariate adjustment in participants. (**a**) Crude model; (**b**) adjusted for education level, marital status, smoking, alcohol consumption, tea consumption, physical activity and heart disease. *MetS* metabolic syndrome, *BMI* body mass index, *FPG* fasting plasma glucose, *HDL* high-density lipoprotein cholesterol, *OR* odds ratio.

In the total study sample, the presence of depressive symptoms was significantly associated with MetS and MAP. Compared with non-depressive counterparts, the presence of depressive symptoms increased odds of MetS, after adjusting for education level, marital status, smoking, alcohol consumption, tea consumption, physical activity and heart disease (OR = 2.53, 95% CI = 1.07, 5.94, *P* = 0.03). Only the relationship between depressive symptoms with MAP (OR = 0.04, 95% CI = 0.002, 0.98, *P* = 0.048) was significant after adjusting for confounding factors rather than in the crude model (Fig. 2).

## Discussion

In this community-based cohort study among older people aged 60 years or over, we found that participants with initial depressive symptoms tended to have increased risk of MetS. Focusing on the individual components of MetS, the presence of depressive symptoms (as a continuous variable) was associated with the change in MAP after adjusting for potential confounders. However, the magnitude of the association was relatively weak. Our findings also provided information for which the interventions targeting lifestyle risk factors could reduce the development of MetS.

In the present study, the presence of depressive symptoms at baseline was associated with approximately twice the odds of having MetS in the full sample; this association remained statistically significant after controlling for the confounding variables. Several studies have linked depression or depressive symptoms with MetS. However, the findings are controversial and most of these studies are cross-sectional design. Our finding is consistent with a cross-sectional analysis among older people in Japan^40^ and a cohort study among middle-aged people in French (65–70 years old at baseline)^41^. It is especially noted that researchers demonstrated opposite results in two of the largest studies in Norway^42^ and Finland^20^, respectively. Nevertheless, some plausible pathophysiological mechanisms have been proposed to help elucidate the association between depression and MetS. Firstly, the hypothalamic-pituitary adrenal axis associated with the clinical depression is activated in MetS, which is related to visceral obesity^43^ and impaired insulin sensitivity^44,45^. Besides, the prospective relationship between initial depressive symptoms and later MetS involves effects of physiological correlates of stress and dysphoric emotion^46^, which also influence the dysregulations mentioned above. Secondly, the dysregulation of the autonomic nervous system via such pathways as aberrant serotonergic functioning^47^ has been proposed as a possible mechanism.

The present results also indicated that the presence of depressive symptoms was inversely associated with the change in MAP. We defined BP as a single measure of MAP, on account of MAP is more accurate in predicting future MetS among the elderly population^37,48^. The association remained negatively significant in logistic regression models. Our finding is in line with prior prospective study investigating the association of depressive symptoms with developing MetS among Iranian elderly population, especially in women^19^. The interaction between depression and hypertension is pathophysiologically reasonable^49^. A meta-analysis of prospective cohort found that patients with depressive and hypertension have increased sympathetic tone and increased secretion of adrenocorticotropic hormone and cortisol^50^. Meanwhile, researchers have proposed a relationship between sympathetic activity and increased BP and emotional problems with MetS^51^. The possible reason for our result may be the side effects of antidepressant use. Some drug therapies have been reported that could negatively affect BP in elderly individuals^52,53^. Moreover, follow-up study has reported that the positive correlation between initial depression and later hypertension may exist during the follow-up for 5 years and longer^54^. More research is encouraged to clarify this relationship.

Although the incidence of MetS was higher among participants with depressive symptoms compared with their counterparts, a 4.86%, 0.69%, and 0.69% of the excess incidence was due to the current smoking, alcohol consumption and history of heart disease on the cohort, respectively. Tea consumption reduced 9.72% of the excess incidence, though did not reach the statistical significance (*P* = 0.05). We think that nowadays tea culture prevails in more than 160 countries, especially in China. This is not sufficient to turn around the onset of MetS worldwide, but the result indicates that tea consumption is negatively associated with MetS. A growing body of evidence suggests that several bioactive components in tea have the potential in adjusting blood lipids^35^ and benefit cardiovascular and metabolic health^55^.

The strengths in this study include its longitudinal design among older people and the use of standard assessment of depressive symptoms. Some limitations of this study should also be noted. First, we measure the depressive symptoms with a symptom scale, which did not reflect the depression as accurately as diagnosis. Second, for living habits, self-report method is adopted, which could lead to information bias (e.g., recall bias and reporting bias). Third, this study focused on whether depressive symptoms at baseline associated with subsequent metabolic syndrome, rather than the changing trend of the association. Finally, our study is based on a community-dwelling population, which the number of depression patients is relatively smaller than that of the clinical population.

In conclusion, this study demonstrated a significant association of the presence of depressive symptoms with MetS and its component in the sample of Chinese elderly. However, hypertension was negatively associated with depressive symptoms when adjusted for confounders. Further research is warranted to investigate the association of BP with depression using large longitudinal cohorts.

## Abbreviations

MetS: Metabolic syndrome
HDL-C: High-density lipoprotein cholesterol
BP: Blood pressure
TG: Triglycerides
FPG: Fasting plasma glucose
PHQ-9: The nine-item patient health questionnaire
MAP: Mean arterial pressure
VIF: Variance infiltration factor
OR: Odds ratio
CI: Confidence intervals
